# Supplementary material for: Coordination of two opposite flagella allows high-speed swimming and active turning of individual zoospores
Source: eLife. 2022 Mar 28;11:e71227. doi: 10.7554/eLife.71227 (PMC9068220; doi:10.7554/eLife.71227)
Supplement: Transparent reporting form [file elife-71227-transrepform1.docx]

***eLife’s* transparent reporting form**

We encourage authors to provide detailed information *within their submission* to facilitate the interpretation and replication of experiments. Authors can upload supporting documentation to indicate the use of appropriate reporting guidelines for health-related research (see [EQUATOR Network](about:blank)), life science research (see the BioSharing Information Resource), or the [ARRIVE guidelines](about:blank) for reporting work involving animal research. Where applicable, authors should refer to any relevant reporting standards documents in this form.

If you have any questions, please consult our Journal Policies and/or contact us: [editorial@elifesciences.org](about:blank).

**Sample-size estimation**

- You should state whether an appropriate sample size was computed when the study was being designed
- You should state the statistical method of sample size computation and any required assumptions
- If no explicit power analysis was used, you should describe how you decided what sample (replicate) size (number) to use

Please outline where this information can be found within the submission (e.g., sections or figure legends), or explain why this information doesn’t apply to your submission:

No explicit power analysis was applied in our study as we did not perform hypothesis testing or comparisons. We study how the two opposite flagella contribute to individual swimming of zoospores without external interactions. 58 individual zoospore trajectories were captured at 60fps in our experiments. Each trajectory has up to 3600 (x,y)-position datapoints.

**Replicates**

- You should report how often each experiment was performed
- You should include a definition of biological versus technical replication
- The data obtained should be provided and sufficient information should be provided to indicate the number of independent biological and/or technical replicates
- If you encountered any outliers, you should describe how these were handled
- Criteria for exclusion/inclusion of data should be clearly stated
- High-throughput sequence data should be uploaded before submission, with a private link for reviewers provided (these are available from both GEO and ArrayExpress)

Please outline where this information can be found within the submission (e.g., sections or figure legends), or explain why this information doesn’t apply to your submission:

We cultured and conducted microscopic assays on several different batches of zoospores. We arbitrarily diluted zoospores so that their concentration was low and few zoospore-zoospore interactions happened as we were investigating the individual swimming of zoospores without any interference. Trajectories encountering zoospore-zoospore interactions were excluded in the data. Videos captured all of them displaying the same qualitative and quantitative behavior.

**Statistical reporting**

- Statistical analysis methods should be described and justified
- Raw data should be presented in figures whenever informative to do so (typically when N per group is less than 10)
- For each experiment, you should identify the statistical tests used, exact values of N, definitions of center, methods of multiple test correction, and dispersion and precision measures (e.g., mean, median, SD, SEM, confidence intervals; and, for the major substantive results, a measure of effect size (e.g., Pearson's r, Cohen's d)
- Report exact p-values wherever possible alongside the summary statistics and 95% confidence intervals. These should be reported for all key questions and not only when the p-value is less than 0.05.

Please outline where this information can be found within the submission (e.g., sections or figure legends), or explain why this information doesn’t apply to your submission:

- We indicated the statistical analysis methods (Mean, Standard Deviation (SD), Standard Error (SE)) right at the presented data. For example, the distribution of *U* in Figure 2d follows a bimodal distribution with the mean *μ* and SD values written in section “Statistics of swimming patterns” and in Figure 2d description.

- All the measurements of zoospore characteristics in SEM, TEM and Bright Field Microscopy were conducted with *N* > 10.

(For large datasets, or papers with a very large number of statistical tests, you may upload a single table file with tests, Ns, etc., with reference to sections in the manuscript.)

**Group allocation**

- Indicate how samples were allocated into experimental groups (in the case of clinical studies, please specify allocation to treatment method); if randomization was used, please also state if restricted randomization was applied
- Indicate if masking was used during group allocation, data collection and/or data analysis

Please outline where this information can be found within the submission (e.g., sections or figure legends), or explain why this information doesn’t apply to your submission:

This doesn’t apply to our work.

**Additional data files (“source data”)**

- We encourage you to upload relevant additional data files, such as numerical data that are represented as a graph in a figure, or as a summary table
- Where provided, these should be in the most useful format, and they can be uploaded as “Source data” files linked to a main figure or table
- Include model definition files including the full list of parameters used
- Include code used for data analysis (e.g., R, MatLab)
- Avoid stating that data files are “available upon request”

Please indicate the figures or tables for which source data files have been provided:

We have published all our experimental data (presented in Figure 2 and 4) via Zenodo, following the DOI: [https://doi.org/10.5281/zenodo.4710633](about:blank). In the published data, we also included MatLab codes to compute all the plots in Figure 2(d-g) and to generate the zoospore spreading simulation in Figure 2(h-i).
